# Parliament and the Profession

**Published:** 1918-06-22

**Authors:** 


### Parliament and the Profession.

### Dental Surgeons.

The Under-Secretary of State for War informed Mr. Pennefather that the number of qualified dental surgeons who hold commissions in the Army is 640; of these, ninety-two have received their commissions since March 1 last, including twenty-six from the ranks. As regards the number remaining in the ranks inquiry is being made.

### Hospitals Bombed by Germans.

Mr. Macpherson informed Mr. Joynson-Hicks that a recent report from the Commander-in-Chief shows that, during the period from May 15 to June 1, hospitals have been bombed on seven occasions. The total casualties in these seven raids were as follows: *Killed*: Officers, 11; other ranks, 218; sisters, 5; Queen Mary's Army Auxiliary Corps, 8; civilians, 6; total, 248. *Wounded*: Officers, 18; other ranks, 534; sisters, 11; Queen Mary's Army Auxiliary Corps, 7; civilians, 23; total, 593; total casualties, 841.

### Alexandra Flag Day.

Colonel Thorne asked the Home Secretary if, in view of the difficulty in obtaining flag sellers in districts like the borough of West Ham, the age limit of sellers could not be reduced from eighteen to sixteen. Mr. Brace said that the rule in question was made by the Home Secretary because he was satisfied that it was not in the interest of young persons under eighteen that they should be allowed to act as collectors in the London streets, and he had no power to make exceptions in special cases, however excellent the objects of the collections might be.

### Leicester Royal Infirmary.

The Minister of Pensions informed Sir Maurice Levy that he had with regret decided that the scheme in connection with the Leicester Royal Infirmary for orthopædic treatment of discharged soldiers, involving the construction of permanent buildings, could not, owing to shortage of labour and materials, be allowed to proceed at the present time. If any permanent building at present in existence could be pointed out the Ministry would be prepared to adapt it for the purpose.

### Postage on Wounded Soldiers' Letters.

The Postmaster-General informed Mr. Tootill that it is regretted that there are serious objections to the grant of free postage to soldiers in hospital in this country. In particular there would be great difficulty in devising effective measures to ensure that the privilege was not taken advantage of by persons not entitled to it.

